# Classifying the Biological Status of Honeybee Workers Using Gas Sensors

**DOI:** 10.3390/s21010166

**Published:** 2020-12-29

**Authors:** Jakub T. Wilk, Beata Bąk, Piotr Artiemjew, Jerzy Wilde, Maciej Siuda

**Affiliations:** 1 Apiculture Division, Faculty of Animal Bioengineering, University Warmia and Mazury in Olsztyn, Sloneczna 48, 10-957 Olsztyn, Poland; beata.bak@uwm.edu.pl (B.B.); jerzy.wilde@uwm.edu.pl (J.W.); maciej.siuda@uwm.edu.pl (M.S.); 2 Mathematical Methods and Computer Science Division, Faculty of Mathematics and Computer Science, University Warmia and Mazury in Olsztyn, Sloneczna 48, 10-957 Olsztyn, Poland; piotr.artiemjew@uwm.edu.pl

**Keywords:** honey bee, gas sensor, electronic nose, decision systems, classification

## Abstract

Honeybee workers have a specific smell depending on the age of workers and the biological status of the colony. Laboratory tests were carried out at the Department of Apiculture at UWM Olsztyn, using gas sensors installed in two twin prototype multi-sensor detectors. The study aimed to compare the responses of sensors to the odor of old worker bees (3–6 weeks old), young ones (0–1 days old), and those from long-term queenless colonies. From the experimental colonies, 10 samples of 100 workers were taken for each group and placed successively in the research chambers for the duration of the study. Old workers came from outer nest combs, young workers from hatching out brood in an incubator, and laying worker bees from long-term queenless colonies from brood combs (with laying worker bee’s eggs, humped brood, and drones). Each probe was measured for 10 min, and then immediately for another 10 min ambient air was given to regenerate sensors. The results were analyzed using 10 different classifiers. Research has shown that the devices can distinguish between the biological status of bees. The effectiveness of distinguishing between classes, determined by the parameters of accuracy balanced and true positive rate, of 0.763 and 0.742 in the case of the best euclidean.1nn classifier, may be satisfactory in the context of practical beekeeping. Depending on the environment accompanying the tested objects (a type of insert in the test chamber), the introduction of other classifiers as well as baseline correction methods may be considered, while the selection of the appropriate classifier for the task may be of great importance for the effectiveness of the classification.

## 1. Introduction

The development of gas sensor technology, including increasing accuracy and affordability, allows research into solutions supporting various areas of economy. Practical beekeeping struggles with many problems related to the functioning of bee colonies. Among the most important factors resulting from bee biology is the presence of the queen bee. One of the disturbances of the normal state of a bee colony is queenlessness, which, when it is long lasting, usually leads to a colony’s destruction. In some cases, the queenless state is difficult to recognize, even by experienced beekeepers. The phenomenon of changing the smell of a bee colony, depending on its biological status associated with the presence of the queen, gives potential for the use of gas sensor technology to support this area of the economy, which is one of the most important sectors of agriculture due to the pollination function.

### 1.1. Biological Status of Worker Bees vs. Odor

It is known that social insects living in large colonies can identify each other. This is due to the specific pheromone profile generated by the mechanism of homogenization and transmission of the cuticular hydrocarbons [1] inside the colony. The honey bee is one such insect. Additionally, its workers play strictly defined, age-dependent roles in the superorganism of a bee colony. Recent studies have shown that the individual profile of the cuticular hydrocarbons of the entire bee colony depends on the quantity and quality of chemical mediators of bees differing in age. Moreover, the cuticular hydrocarbon profile of individual workers of honeybees is also associated with age-related role distribution [2].

The link that integrates a bee colony with the pheromone of the maternal substance is the queen [3]. She is the only female in the bee colony with a fully mature reproductive system; because of this, she can mate with drones and lay fertilized eggs from which female specimens—workers—develop. Male individuals develop from unfertilized eggs.

A special condition of a bee colony is the appearance of physiological laying worker bees (worker bees that lay unfertilized eggs). The reason for this unfavorable phenomenon is the long-term absence of a queen in the bee colony. As a result, there is a deficiency of the queen substance, which normally inhibits the development of the reproductive system in workers [4]. In a queenless colony, some workers develop swollen ovaries, and after some time, 5–24% of workers start laying eggs. However, worker bees are not capable of mating, so these eggs are unfertilized and they develop into drones, which is why workers who produce them are called physiological laying worker bees. This abnormal condition is characterized by the increased amount of drone brood present in the honeycomb cells. However, until the 10th day after laying the egg, before the brood comb cell is sealed, it is difficult to distinguish such brood from normal bee brood [5].

Colonies with laying worker bees find it difficult to undergo emergency procedures aimed at rearing a new queen. This can lead to the destruction of the bee colony.

### 1.2. The Context of the Problem in Practical Beekeeping

During the beekeeping season, problems arise with colonies where physiological laying worker bees lay eggs. Beekeepers may find it difficult to distinguish a queenless colony in which laying worker bees started the process of laying eggs from a colony that is functioning properly, or one in which the queen has become a laying worker bee, i.e., has lost the ability to lay fertilized eggs. Individual physiological laying worker bees cannot be distinguished with the naked eye from an ordinary bee worker. The beekeeper is therefore unable to identify their presence. Laying worker bees’ eggs are often laid several in one bee cell or on the sidewalls of the cell. These are characteristic symptoms, but do not always denote the existence of physiological laying worker bees who lay eggs. The presence of unfertilized eggs in the combs can sometimes confuse even experienced beekeepers, who mistakenly believe that they are dealing with the eggs of a queen. Difficulties in proper assessment of the colony’s status will delay rescue procedures, leading to increased losses in the apiary.

In a properly functioning bee colony without a queen, workers can rear a new queen, a so-called emergency queen. It arises from 1–3 day-old bee larvae, which hatches from fertilized eggs previously deposited by the lost queen. This must take place within 6 days.

Even when gradually emerging laying worker bees begin to lay eggs, workers will not be able to rear a new queen from them, because these eggs are unfertilized and form drone larvae. This situation leads to the destruction of the bee colony, as a result of the gradual extinction of old workers. Although there is a chance to rear a queen from an unfertilized egg [6,7], it is so small that it is negligible from the practical point of view of beekeeping.

For the reasons described, the condition of the colony should be constantly monitored for the presence of a properly reproductive queen. The type and effectiveness of emergency procedures depend on the negative consequences of a queenless colony, which increases over time.

### 1.3. Achievements to Date in the Use of Gas Sensors for this Type of Task

The electronic nose is a device that detects unidentified, complex substances. It is made up of a matrix of partially sensitive chemical sensors capable of recognizing simple or complex odors on the basis of a pattern recognition system [8]. The use of the electronic nose is one of the methods based on a comprehensive analysis of the volatile fraction without its division into individual components. This distinguishes it from methods based on the analysis of separated sample components, such as chromatography techniques. It does not require a time-consuming process of sample preparation, which also is usually destroyed [9]. In addition to the selection of sensors, the basis for the method is the appropriate analysis of measurement data, which can lead to the most effective classification.

There are many methods of analysis in the field of using the electronic nose in beekeeping, including linear discriminant analysis (LDA), principal component analysis (PCA), and cluster analysis (CA) with the furthest neighbor method (kNN). Good results have also been obtained using the artificial neural network (ANN) machine learning techniques, which use a neural network model based on a multilayer perceptron that learned using a backpropagation algorithm [8,9,10,11,12,13,14,15].

A related study found that the odor profile of the entire colony changed depending on the degree of infestation of the bee parasite Varroa destructor mite [16]. In this case, the gas sensors reacted to samples of the hive air taken directly from the bee colony being tested, constituting its general odor in the nest. Classification according to the degree of parasite infestation was successfully performed [10]. Rather than using one type of sensor, an electronic nose was employed. The sensor matrix was also successfully used to quickly recognize five different types of honey collected in Poland [9]. Other attempts to classify honey according to its type and origin also gave good results [12,13].

## 2. Materials and Methods

### 2.1. Characteristics of the Studied Objects

The samples were obtained from the *Apis mellifera carnica* bee colonies situated in the experimental apiary of the Department of Apiculture (University of Warmia and Mazury in Olsztyn, Poland), coordinates 53°44′41.8″ N 20°26′57.2″ E. Three classes of objects (samples) from bee colonies were used:

- Young workers—class 5
- Old workers—class 6
- Workers from colonies with physiological laying worker bees—class 7 (Table 1)

Additionally, an empty test chamber was tested as a control class—class 1.

**Figure 1:**
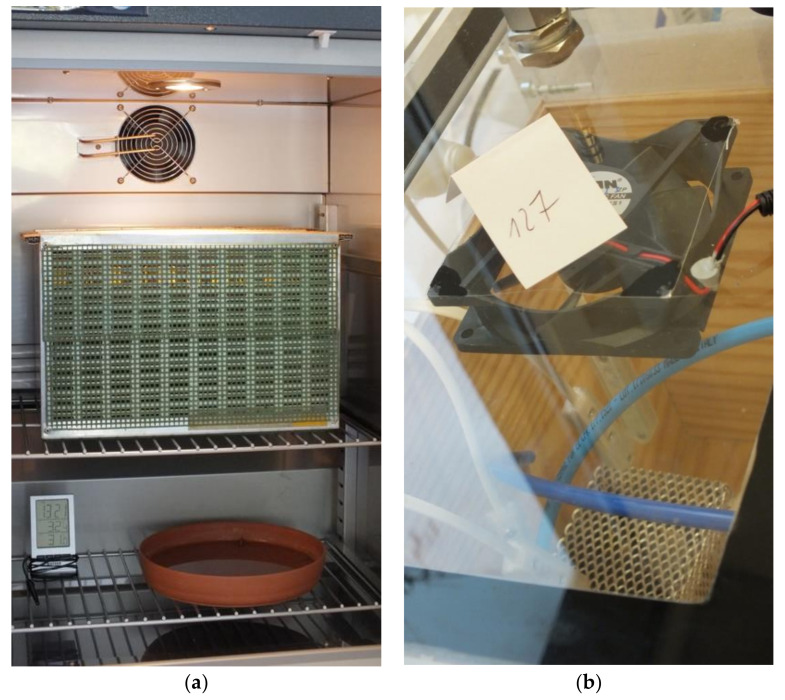
Object preparation and examination—young bees. (**a**) The sealed brood comb was enclosed in an insulator and placed in an incubator awaiting emergence of the bees. (**b**) An aluminum cage with the young bees (100 pcs) who emerged during the test in the test chamber (in this case, a chamber with a wooden insert).

Young worker bees were obtained from sealed brood collected from six bee colonies on 18 and 23 October 2018. The brood honeycombs were placed in an incubator in an insulator to prevent divergence of bees and were kept at 35 °C until the bees emerged. As many as 100 emerged bees, 0–1 days old, were counted for each trial and placed in an aluminum mesh cage (Figure 1). In this way, 10 samples of young bees were prepared.

Old worker bees originated from the outer nest combs (not covered by brood) from six bee colonies. The samples were taken on 17 and 18 October 2018. After the CO_2_ anesthesia, 100 bees were counted for each test and placed in an aluminum mesh cage. Before testing, the samples were kept in an incubator at 35 °C for a minimum of 1 h to calm the bees. As many as 10 samples were prepared this way.

**Figure 2:**
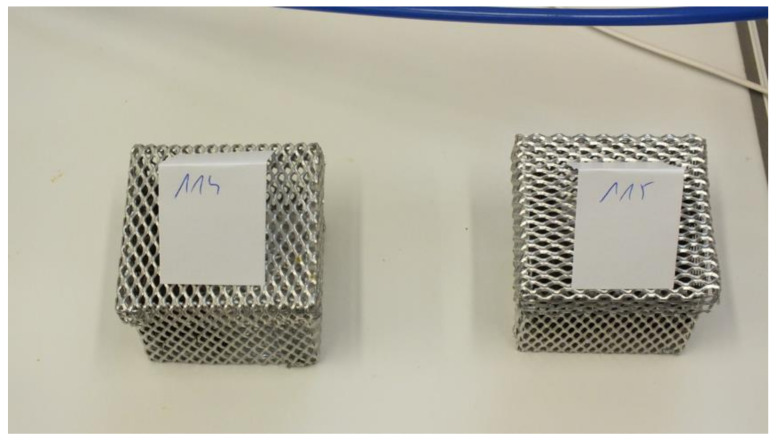
Aluminum mesh cages with workers (100 pcs each) from colonies with physiological laying worker bees.

Workers from colonies with physiological laying worker bees originated from honeycombs taken from two long-term queenless colonies in which the presence of laying worker bees’ eggs, drone brood, a large number of drones, and no worker bee brood were found. The samples were taken on 24 and 25 September 2018 and on 4, 5, and 23 October 2018. After the CO_2_ anesthesia, the drones were collected and 100 bees were counted and placed in an aluminum mesh cage. As many as 10 samples were prepared this way (Figure 2).

### 2.2. Construction of a Laboratory Measuring Stand—Multi-Sensor Signal Recorder

**Figure 3:**
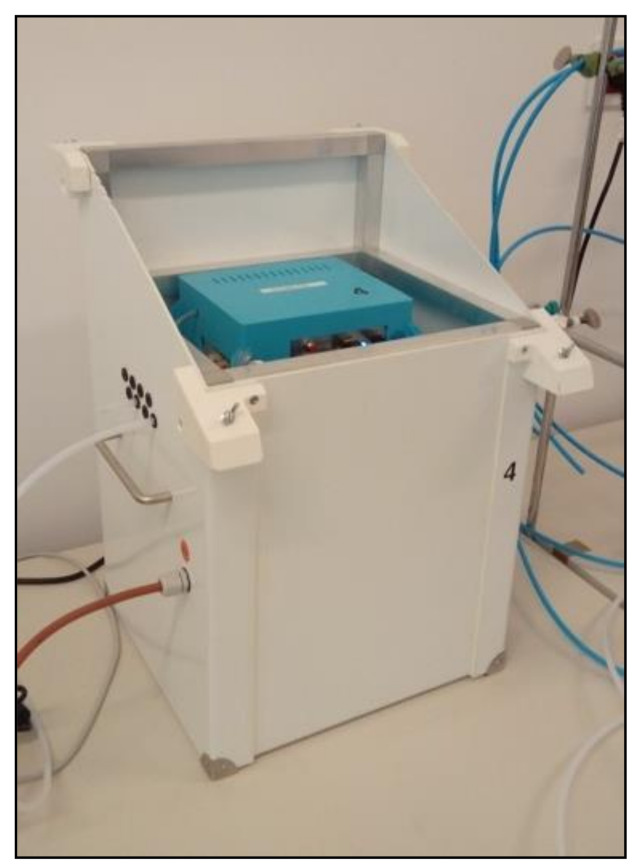
Gas sensor device—general view.

**Figure 4:**
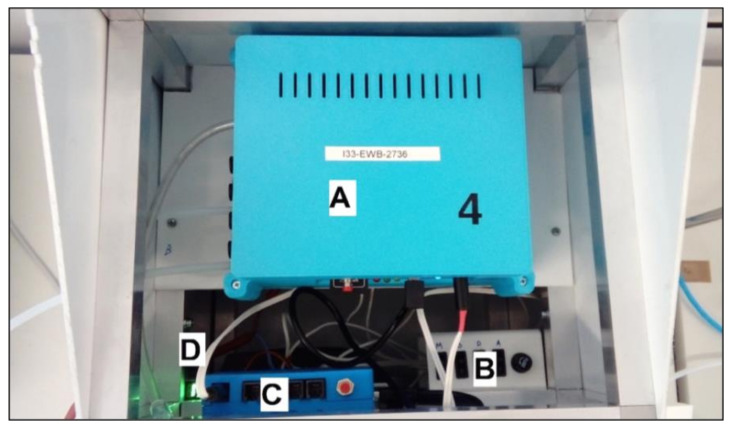
Gas sensor device—main functional modules: (**A**) multi-sensor device MCA-8, (**B**) charge regulator, (**C**) communication controller, and (**D**) battery charge level indicator.

The research used a prototype device constructed in the Laboratory of Sensor Technique and Indoor Air Quality Studies at Wroclaw University of Science and Technology. It was a multi-sensor, multi-channel signal recorder (Figure 3). The sensor matrix integrated into the module (named MCA-8) was made of six Figaro semiconductor gas sensors (Figure 4A).

Sensors commonly available for purchase were selected (Table 2). Each of the sensors placed in the matrix was led by an individual gas input and output, as well as appropriate electrical connections. The device was characterized by a dynamic method of sampling the tested gas, which was supplied to the matrix through a program-selected channel with the use of a pump. After passing through the separate chambers of each sensor, the gas was exhausted to the output of the device. The device had eight sequentially working channels creating separate gas paths to the device matrix. The operation of the device was controlled by reading the parameters from the configuration file recorded on the SD card, which made it possible to set the time and sequence of individual channels, heater power, number of repetitions, and pump power. The desired control of the gas path was carried out by valves.

Measurement results were recorded on an SD card and sent via the GSM (Global System for Mobile Communications) communication module (Figure 4C) to a dedicated server in the form of structured CSV (comma-separated values) files. A single measurement point was taken every 1 s and was the average reading of the measurement in a given second; this measurement was taken separately on each of the sensors. The device was powered by 220V mains voltage; however, it can also be run on the built-in 12V battery. The battery is charged from the mains or with a set of solar panels using the charge regulator control (Figure 4B).

### 2.3. Construction of a Laboratory Measuring Stand—Resarch Chamber

**Figure 5:**
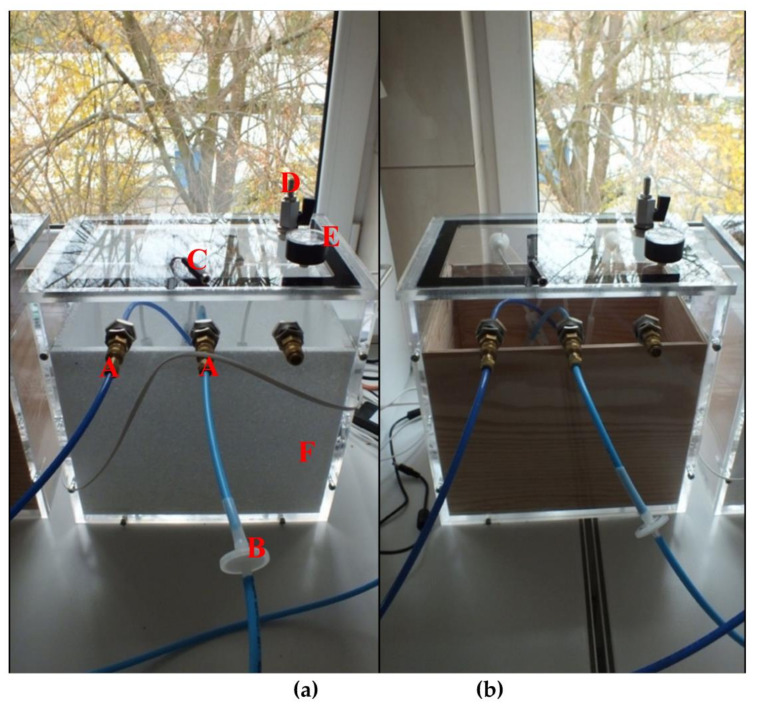
(**a**) Test chamber with a polystyrene insert. (**A**) Two gas lines to the measuring devices formed by a pneumatic connection. (**B**) Particulates filter. (**C**) Visibly disconnected power from the mechanical fan, which was not used during the tests. (**D**) Air inlet to the chamber with a regulator. (**E**) Pressure gauge. (**F**) Polystyrene insert placed in the chamber. (**b**) Identical test chamber with a wooden insert.

For the laboratory tests of hive environment objects, special research chambers were built using measuring instruments (Figure 5).

The chambers, measuring 32 × 22 × 32 cm, were made of Plexiglas. Each was equipped with three pneumatic connectors constituting an element of the gas track, three sealed visors for inserting any sensors, flexible plugs for interchangeable use for each visor (full and notched—sealing the introduction of, e.g., sensor cables), a fan equalizing the level of volatile substances from the tested object in the entire volume of the chamber, and an adjustable air supply valve.

Two inserts for the research chamber were prepared: polystyrene and wooden. The inserts reflected the properties of the materials of which the hives are built (wood, polystyrene).

The construction of the chamber met the following assumptions:

- a tight structure, aimed at eliminating factors that may interfere with the odor of the tested objects,
- small dimensions, maximizing the concentration of volatile substances around the sensors,
- adjustable tightness of test chambers in terms of the possibility of external gas inflow,
- using neutral, odorless materials to build the chambers, with removable inserts,
- the possibility of measuring the pressure in the chamber,
- the possibility of mechanical equalization of the level of volatile substances from the tested object in the entire volume of the chamber through use of the fan, and
- the possibility of simultaneous measurement of a given object with at least two devices.

### 2.4. Measurement Procedure

The measurements were carried out in the Laboratory of Bee Products Quality at the Department of Apiculture at the University of Warmia and Mazury in Olsztyn. Two twin measuring devices, called M1 and M2, were used simultaneously. Before starting the first measurements on a given day, the device was heated up; it ran for a minimum of 1 h at 100% of the heater’s power, without the pump running (pump power 0%). Following this, measurements were carried out continuously on a given day to prevent the device from cooling down. The study was carried out on one of eight working channels (1–8). The working channel was changed after every two days of work. Object measurements were made at room temperature with both devices simultaneously.

The tested objects were placed in the research chamber. Directly above the tested object, at a distance of 2–3 cm, the tip of the pneumatic conduit was introduced to collect a gas sample above the object. The gas was sucked dynamically through a working pump of the device and polyethylene (PE) pipes introduced through sealed pneumatic joints (Figure 5A). The gas was sent to the chamber of the sensory matrix of the device. In addition, the filter was placed in the gas track to retain solid particles (Figure 5B). The sucked air was replenished into the chamber freely from the ambient air through the fully open inlet valve (Figure 5D). The gas sampling procedure was not to cause changes in the atmospheric pressure in the test chamber. During all measurements, the pump was running at 50% power.

The measurement sequence of a given object consisted of the 10 min phase of exposure to the test object placed in the chamber. Immediately after exposure to the tested object, the device was switched to the sensor regeneration phase, following the set configuration. Channel 8 was always used; the air was drawn through the carbon filter for the same time, with the same power of the pump and heaters. Each object was tested twice; immediately after the end of the measurement in the polystyrene chamber, the object was transferred to the chamber with a wooden insert and a new measurement of the same object was started.

The single described phase of exposure to an object and the subsequent regeneration phase together formed the measurement of one object, during which the measurement signal reading was recorded every second. This gave a total of 600 readings during the exposure phase and 600 readings during the sensor regeneration phase. This resulted in a total of 1200 readings for one full measurement of an object in the chamber with a polystyrene insert and 1200 readings for a full measurement of the object in the chamber with a wooden insert for each of the devices.

### 2.5. Data Analysis Procedure

**Figure 6:**
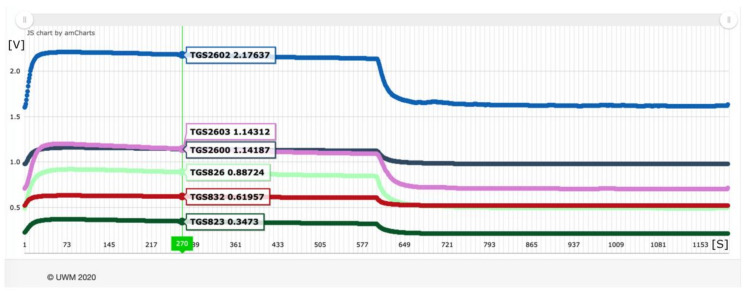
An example of a line graph of the average reading from all class 6 objects for individual sensors. The safety of stabilizing the measurement signal ensures the 270 s of measurement.

The measurement data collected was analyzed. In the first step, the data in the prepared visualization system was reviewed. Based on the observation of line charts from sensor readings, no disturbances were found in the course of measurements of individual objects, and rapid stabilization of the signal was noted. Based on the observation of the average reading graphs for the objects in a given group for individual sensors, 270 s of the measurement were arbitrarily determined, after the signal stabilization, as a reference for further analyses (Figure 6).

The results were analyzed before and after the baseline adjustment. The baseline correction improved the separability of the tested objects within the classes in previous research in the field of beekeeping, based on the same type of sensor matrices [6,7]. The baseline differential correction was applied by subtracting from the value of the measurement of the tested object in 270 s the values from the last 600 s of the ambient air measurement. The results for the objects tested in chambers with both types of inserts (wooden and polystyrene) were analyzed and compared separately for both devices.

The analyses aimed to investigate the differences in the responses of gas sensors exposed to air from worker bees with different biological status according to the assumed class division and type of insert in the chamber in which the measurement was performed (wooden or polystyrene) and to indicate an effective method of classification of the tested objects. In beekeeping practice, the presence of physiological laying worker bees in the bee colony creates a problem; therefore, special attention was paid to class 7 objects.

The possibility of recognition was tested, which consists of assigning a class to the tested object. Classification consists of a decision on which class the tested object can be assigned. This task was performed using classifiers constructed as follows.

In basic research, a five-fold cross-validation 2 test was performed. The nearest neighbor (kNN) algorithm with the default settings of the rough set exploration system (RSES) tool with automatically selected k was chosen as the classifier.

Following this, customized techniques dedicated to the task were used. Studies with dedicated methods were carried out according to the Monte Carlo cross-validation (MCCV) model, using the same subsets of data for all techniques studied. Conditional attributes used in the research were numerical, while decision classes were categorical; hence, we applied a Naïve Bayes classifier with a descriptors indiscernibility ratio [17], the kNN method with Euclidean, Manhattan, and Canberra metrics [17], and the weighted voting classification technique—algorithm 811 [17]. To correctly estimate the effectiveness of unbalanced data, we used balanced accuracy (the equivalent of the recall parameter) and true positive rate (the equivalent of the precision parameter). Where more than two classes were classified, each of these parameters was calculated separately for each class. Balanced accuracy is the average accuracy of the classification from all decision classes, while the accuracy of classification of a given test class is understood as the percentage of correctly classified objects in that class. True positive rate is the percentage of objects of a given class positively classified in relation to all objects classified in that class. The coverage parameter is the percentage of classified objects. In our case it was always equal to one; in other words, each test object was classified.

## 3. Results

### 3.1. The Visualization of the Tested Classes

**Figure 7:**
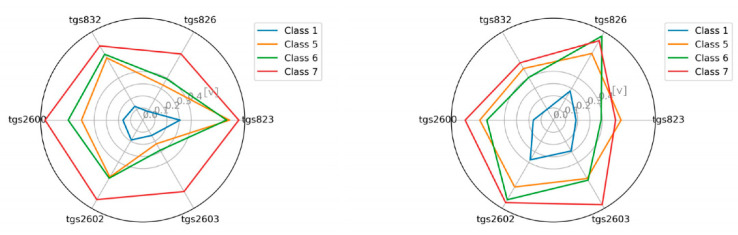
The visualization of the average reading intensity of TGS sensors for decision classes 1, 5, 6, and 7 using measuring device M1; wooden chamber on the left, polystyrene chamber on the right.

**Figure 8:**
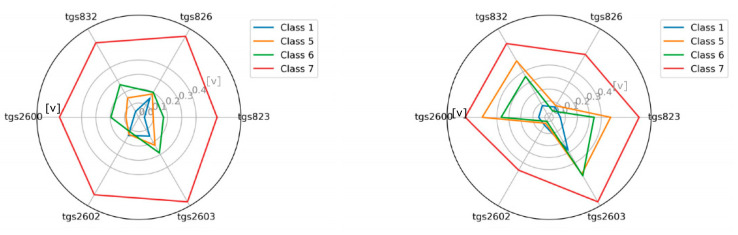
The visualization of the average reading intensity of TGS sensors for decision classes 1, 5, 6, and 7 using M1 with baseline correction; wooden chamber on the left, polystyrene chamber on the right.

**Figure 9:**
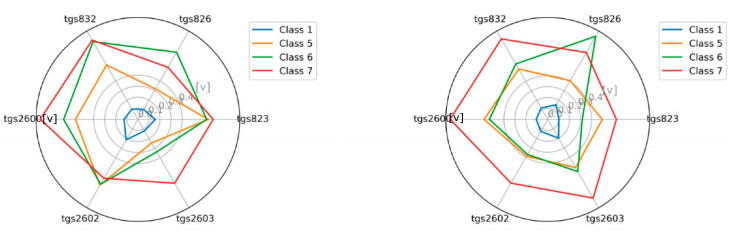
The visualization of the average reading intensity of TGS sensors for decision classes 1, 5, 6, and 7 using measuring device M2; wooden chamber on the left, polystyrene chamber on the right.

**Figure 10:**
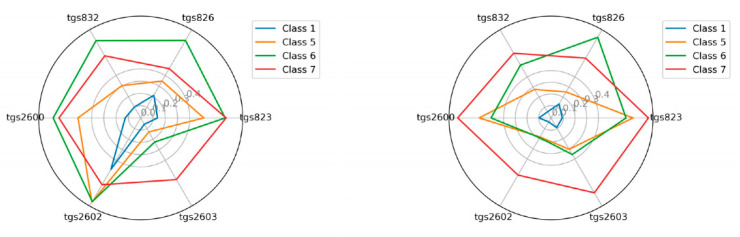
The visualization of the average reading intensity of TGS sensors for decision classes 1, 5, 6, and 7 using M2 with baseline correction; wooden chamber on the left, polystyrene chamber on the right.

As an additional element, we attempted to visualize the sensor readings for individual classes in the radar chart (Figure 7, Figure 8, Figure 9 and Figure 10). This is akin to a simple scent signature. To achieve this goal, we normalized the values of the TGS attributes to a range of [0.1] using Equation (1). Normalization of TGS_i_(ob_j_) (i-th descriptor of j-th object) into [a, b] interval consists of the following step:

(1) $$TGS_{i}\left(ob_{j}\right)=\frac{\left(TGS_{i}\left(ob_{j}\right)-min_{TGS_{i}}\right)\;*\;\left(b-a\right)}{max_{TGS_{i}}-min_{TGS_{i}}}+a$$

Then we calculated the average values in the classes for the individual TGS attributes (see Equation (2)) and squared the obtained values (see Equation (3)), as follows:

(2) $$average\left(TGS_{i}^{class_{j}}\right)=\overset{\left|class_{j}\right|}{\underset{k=0}{\sum }}TGS_{j}\left(ob_{l}\right),\;where\;ob_{l}\in class_{j}$$

(3) $$average\left(TGS_{i}^{class_{j}}\right)=average{\left(TGS_{i}^{class_{j}}\right)}^{2}$$

These results clearly show that classes are potentially different and separable.

### 3.2. Searching for the Dedicated Method—Selected Results

We presented our implementation using the multiple MCCV model. MCCV is based on multiple executions of the train and test method, with a defined random percentage, split into training and test parts. In search of an effective, simple classification method, we implemented the Naive Bayes classifier [14], the weighted classification 811 method [14], and the local kNN using Manhattan, Euclidean, and Canberra metrics [14]. Naive Bayes uses sum instead of multiplication to avoid zeroing of parameters and descriptors’ indiscernibility ratio.

We considered the following test options, using two devices (M1 and M2):

(I) M1 device in a wooden box,
(II) M1 device in a polystyrene box,
(III) M2 device in a wooden box,
(IV) M2 device in a polystyrene box,
(V) M1 device in a wooden box using baseline correction,
(VI) M1 device in a polystyrene box using baseline correction,
(VII) M2 device in a wooden box using baseline correction, and
(VIII) M2 device in a polystyrene box using baseline correction.

We considered the following variants:

- all classes together,
- 1 vs. 5,6,7,
- 5 vs. 1,6,7,
- 6 vs. 1,5,7, and
- 7 vs. 1,5,6.

We considered the following classifiers:

1. canberra.1nn,
2. canberra.811,
3. eps = 0.01.nb,
4. eps = 0.01.nb2,
5. euclidean.1nn,
6. euclidean.811,
7. manhattan.1nn,
8. manhattan.811,
9. maxminnormalized.1nn, and
10. maxminnormalized.811.

**Figure 11:**
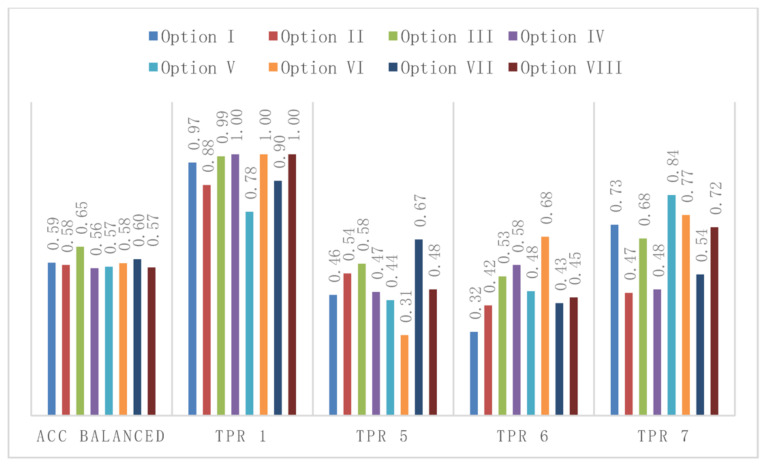
Best classifiers: parameter values in individual options, M1 device. Four-class problem—results of class separation. Values of the Acc balanced and true positive rate parameters, each class, depending on the option for the best classifiers (indicated in the Table 3), M1 device.

Table 3 and Figure 11 presents the results of classifiers operation depending on the options and variants for the four-class problem, based on the accuracy balanced and true positive rate parameters, and taking into account the following calculations:

- Accuracy balanced, the best classifier is m5, in five options, reaching the value of 0.646 for Option III,
- True positive rate for class 1, the best classifier is m2, in four options, in three options reaching the value of 1,
- True positive rate for class 5, the best classifiers are m5 and m10 *pari passu*, each in two options, while the m1 classifier reaches the highest value of up to 0.674 in Option VII,
- True positive rate for class 6, the best classifiers are m4 and m5 *pari passu*, each in two options, while the m5 classifier reaches the highest value of up to 0.684 for Option VI, and
- True positive rate for class 7, the best classifier is m5 in three options, with m10 reaching the highest value of 0.844 for Option V.

The best results in the four-class problem are given by the use of the m5 classifier, which produces the highest values in five options for accuracy balanced, reaching a value of up to 0.646 in Option III. In the case of the true positive rate class 7 parameter, the m5 classifier is also the best for the greatest number of options (3/8), reaching a value of up to 0.73 in Option I.

The results of the classifiers related to class 7 for variant 7 vs. 1, 5, 6 were compared. All options for the M1 device were selected for presentation.

The data in Table 4, Table 5, Table 6 and Table 7 correspond to Options I, II, V, and VI, respectively. In the case of type 1 vs. all test for class 7 (7 vs. 1, 5, 6), based on the results presented (Table 4, Table 5, Table 6 and Table 7), large discrepancies in the values of the parameters depending on the classifier used can be observed. Depending on the option, the largest discrepancies in the true positive rate 7 parameter between the best and the worst classifiers ranged from 0.307666 in Option VI to 0.410713 in Option I, and in the case of accuracy balanced, from 0.131192 in Option I to 0.208876 in Option II.

For Option I, the accuracy balanced parameter had values from 0.631352 (m3 classifier) to 0.762544 (m5 classifier), and the true positive rate parameter for class 7 had values from 0.331623 (m4 classifier) to 0.742336 (m5 classifier).

**Figure 12:**
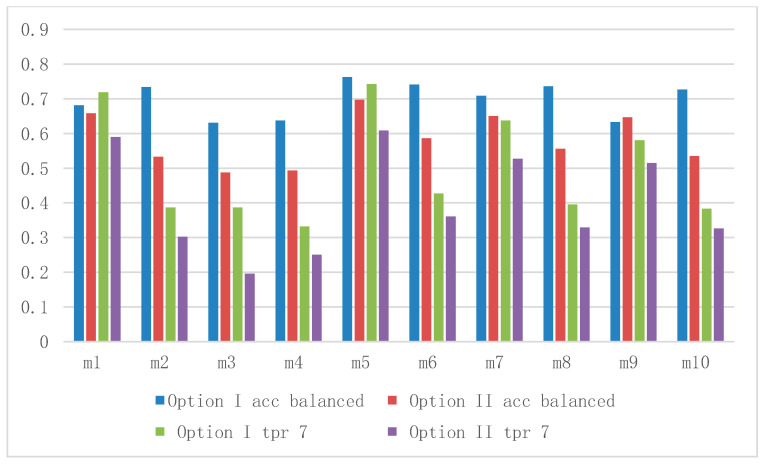
Comparing of average classification result from 25 tests, 5xMCCV5 experiment, 7 vs. all, M1 device, wooden (Option I) and polystyrene (Option II) chambers, all classifiers, Accuracy (acc) balanced, true positive rate 7 (tpr7). We can see that in Option I the parameters reach higher values. The m5 classifier is best for both options.

For Option II, the accuracy balanced parameter had values from 0.487912 (m3 classifier) to 0.696788 (m5 classifier), and the true positive rate parameter for class 7 had values from 0.195856 (m3 classifier) to 0.608952 (m5 classifier). A comparison of the results of the key parameters of Options I and II for all variants is shown in Figure 12. Significantly better results are observed for the wooden box (Option I).

For Option V, the accuracy balanced parameter had values from 0.6088 (m3 classifier) to 0.76078 (m8 classifier), and the true positive rate parameter for class 7 had values from 0.341942 (m4 classifier) to 0.710188 (m5 classifier).

**Figure 13:**
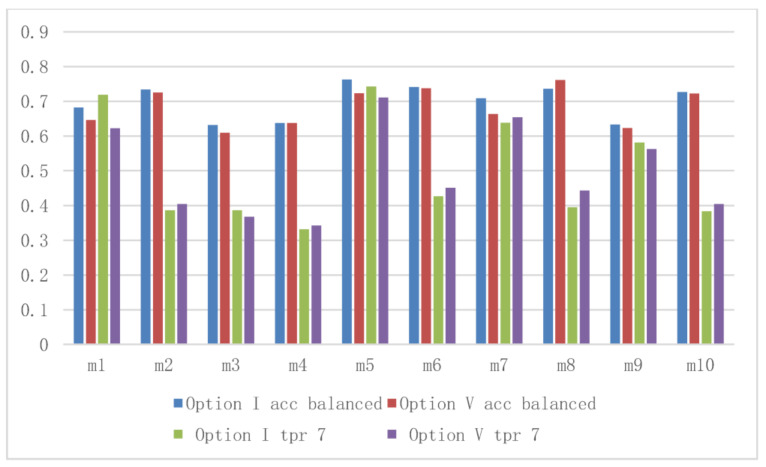
Baseline correction. Comparing of average classification result from 25 tests, 5xMCCV5 experiment, 7 vs. all, M1 device, wooden chamber with (Option V) and without (Option I) baseline correction, all classifiers, accuracy (acc) balanced, true positive rate 7 (tpr7). We can see that in Option I, the accuracy balanced parameter has higher values (except for the m8 variant) and the tpr7 parameter has higher values in 4 variants, so baseline correction decreased the accuracy balanced value, but increased the tpr7 value. However, the m5 classifier, which is best for both options, gives better results without baseline correction (Option I).

For Option VI, the accuracy balanced parameter had values from 0.540308 (m3 classifier) to 0.74224 (m10 classifier), and the true positive rate parameter for class 7 had values from 0.342526 (m4 classifier) to 0.650192 (m6 classifier). A comparison of the results of the key parameters of Options I and V for all variants is shown in Figure 13. Generally better results can be observed for option without baseline correction (Option I).

Tests 1 vs. all performed for each of the classes in all options showed that the highest value of the parameter true positive rate = 1 was obtained in the test of classes 1 vs. 5, 6, and 7 for Options IV and VIII for several different classifiers (Table 8). In the test of class 7 vs. 1, 5, and 6, which is the most interesting for us, the highest value of true positive rate = 0.742 was obtained for Option I using the m5 classifier.

In a similar comparison for the accuracy balanced parameter (Table 9), the highest value of 0.964 was obtained in the test class 1 vs. 5, 6, and 7 for Option VIII using the m1 classifier. In the test of class 7 vs. 1, 5, and 6, the highest value of true positive rate = 0.742 was obtained for Option I using the m5 classifier.

We found that m5, euclidean.1nn, was most often the best classifier for 7 vs. 1, 5, and 6 among all the options. Thus, for this classifier, for the class 7 distinction task (7 vs. 1, 5, 6), Table 10 summarizes the results of the accuracy balanced and true positive rate parameters in all options, including device (M1, M2), type of insert in the chamber (wooden, polystyrene) and use of a baseline correction (without baseline correction, with baseline correction). The highest values of accuracy balanced (0.763) and true positive rate (0.742) were obtained in Option I.

To assess the validity of introducing the baseline correction method, Options I, II, III, and IV were respectively compared in pairs with Options V, VI, VII, and VIII. The only difference between these pairs was the baseline correction factor. We found that the baseline correction increases the effectiveness of the classification in both calculated parameters (accuracy balanced and true positive rate class 7) only for the M2 device with the use of a polystyrene chamber (Option VIII). In other cases, it caused deterioration of at least one of the parameters (Table 10).

In the case of accuracy balanced and true positive rate in test 7 vs. 1, 5, and 6, in individual options for the m5 method, the best results were achieved for both devices in the option with a wooden chamber (Options I and V). The baseline correction increased the efficiency of the classification in both calculated parameters (accuracy balanced and true positive rate class 7) only in the case of the M2 device with the use of a polystyrene chamber. In other cases, it caused deterioration of at least one of the parameters (Table 10).

## 4. Discussion

We can see that the results of the research indicate a profit in distinguishing classes, greater than the standard deviation and considering the expected value of random classification.

Considering that, with all the variants, there are 400 tests to perform, we specify the best results and techniques, as follows.

### 4.1. Four-Class Problem

Without using the baseline correction (Options I–IV), the m5 classifier worked best (it was the best twice), and after its introduction, m6 was the best (it was the best twice). However, globally, for all options and parameters (accuracy balanced, true positive rate for classes 1, 5, 6, 7), the m5 classifier most often appeared as the best; therefore, for this type of task, the m5 classifier is recommended.

In the chamber with a wooden insert (Options I, III, V, and VII), m5 turns was the best (it was the best twice), and in the chamber with a polystyrene insert (Options II, IV, VI, and VIII), m6 was the best (it was the best three times). Therefore, in the four-class problem, the selection of classifiers should be considered depending on the environment in which the tested object is placed. However, when the interest is limited to distinguishing class 7, the m5 classifier should be used as it yielded the best results for both types of chamber without baseline correction.

### 4.2. Choosing the Best Classifier to Distinguish Class 7

In the case of testing 7 vs. 1, 5, and 6, which has the best chance of implementation due to the potential of solving a defined problem occurring in practical beekeeping (detection of colonies with physiological laying worker bees), the m5 classifier was the best in seven out of eight options for the true positive rate parameter, which had a value of up to 0.742 for Option I (M1 device in a wooden chamber) and in five of eight options for the accuracy balanced parameter, the value of which reached 0.763 (also for Option I). One notable finding was that the options introducing potentially more odor noise from the insert material in the chamber (wooden insert, Options I, III, V, and VII) gave higher parameter values.

In summary, without building decision trees for the selection of the classifier depending on a given detailed problem, the m5 classifier is recommended. It was the most appropriate for the defined problem of class 7 differentiation, with the best results in Option I (wooden chamber without baseline correction). The highest results achieved, a true positive rate of 0.742 and an accuracy balanced value of 0.763 in the test 7 vs. 1, 5, and 6 for Option I, are promising and can be used in practical beekeeping. It is particularly noteworthy that wood, a popular hive construction material, does not interfere with the results in a way that would prevent class distinctions.

For the recommended variant of the m5 classifier, the baseline correction for the true positive rate and accuracy balanced parameters brought mixed results, and ultimately showed that no baseline correction should be made. We found that baseline correction, which in previous studies brought good results, should, in the case of the given problem and the selected classifier, be rejected or used only in variants for the polystyrene chamber, where it gave a slight improvement in results.

It is worth noting that there are significant differences in the accuracy of classification between the M1 and M2 devices. As both devices, constructed similarly, were used to measure the same objects at the same time, the difficulty of achieving repeatability in devices where sensors of the type used are the most important element, was confirmed. To achieve proper operation, individual calibration of each device is required.

## 5. Conclusions

The research proved that, with a device using gas sensors and based on testing a sample of 100 bees in the test chamber, the biological status of bees, specifically those from colonies with physiological laying worker bees, can be distinguished. The effectiveness of their differentiation from other classes (bees with a different biological status), determined by the parameters of accuracy balanced and true positive rate, which reached 0.763 and 0.742 in the case of the best euclidean.1nn classifier, may be satisfactory in the context of practical beekeeping. Depending on the environment accompanying the tested objects (the type of insert in the test chamber), the introduction of other classifiers and baseline correction methods can be considered; the selection of the appropriate classifier for the task may be of great importance for the effectiveness of the classification. Due to the differences between devices in the achieved key parameters, the potential problem of calibration deserves special attention.

## Figures and Tables

**Table 1:** Examined objects and their source of origin, i.e., the colony and the date of examination.

Class 5—Young Workers			Class 6—Old Workers			Class 7—Workers from Colonies with Physiological Laying Worker Bees		
Object No.	Object Origin (Colony ID)	Test Date	Object No.	Object Origin (Colony ID)	Test Date	Object No.	Object Origin (Colony ID)	Test Date
106	38	18 October 2018	96	51	17 October 2018	18	10	24 September 2018
108	68	18 October 2018	97	51	17 October 2018	19	10	25 September 2018
110	51	18 October 2018	98	51	17 October 2018	20	10	25 September 2018
111	322	18 October 2018	99	51	17 October 2018	22	10	25 September 2018
112	68	18 October 2018	100	38	17 October 2018	68	66	4 October 2018
125	44	23 October 2018	101	68	17 October 2018	69	66	4 October 2018
126	73	23 October 2018	102	322	17 October 2018	77	66	5 October 2018
127	73	23 October 2018	103	322	17 October 2018	78	66	5 October 2018
128	73	23 October 2018	105	38	18 October 2018	114	66	23 October 2018
129	68	23 October 2018	107	44	18 October 2018	115	66	23 October 2018
			109	68	18 October 2018			

**Table 2:** The measurement characteristics of TGS (Taguchi Gas Sensor) semiconductor gas sensors installed in the gas sensor device (https://www.figaro.co.jp/en).

Sensor	Substances Detected	Detection Range	Heater Power Consumption [mW]
TGS 823	Organic solvent vapors	50 ppm~5000 ppm Ethanol, n-Hexane, Benzene, Acetone	660
TGS 826	Ammonia	30 ppm~300 ppm Ethanol, Ammonia, Isobutane	835
TGS 832	Chlorofluorocarbons	100 ppm~3000 ppm R-407c, R-134a, R-410a, R-404a, R-22	835
TGS 2600	Gaseous air contaminants	1 ppm~100 ppm	210
TGS 2602	VOCs and odorous gases	1~30 ppm Ethanol, Ammonia, Toluene	280
TGS 2603	Amine-series and sulfurous odor gases	1 ppm–30 ppm Ethanol 0.1 ppm–3 ppm Trimethylamine, 0.3 ppm–2 ppm Methyl mercaptan	240

**Table 3:** Four-class problem—results of class separation, 5xMCCV5—average classification results from 25 tests: all classes, M1 and M2 devices, wooden and polystyrene chambers, with and without baseline correction (Options I–VIII). Note: Tpr 1 = true positive rate class 1, Tpr 5 = true positive rate class 5, Tpr 6 = true positive rate class 6, and Tpr 7 = true positive rate class 7.

		Option I	Option II	Option III	Option IV	Option V	Option VI	Option VII	Option VIII	Best Classifier/ Parameter
**Acc _balanced_**	**Classifier**	m5	m5	m5	m7	m5	m5	m1	m6	m5
	**Value**	0.585	0.576	0.646	0.564	0.57	0.583	0.598	0.567	
**Tpr 1**	**Classifier**	m6	m9	m2	m2, m6, m8, m10	m2	m10	m1	m2, m6, m8	m2
	**Value**	0.969	0.883	0.993	1	0.78	1	0.899	1	
**Tpr 5**	**Classifier**	m7	m5	m10	m10	m5	m6	m1	m8	m5, m10
	**Value**	0.462	0.544	0.581	0.473	0.442	0.308	0.674	0.483	
**Tpr 6**	**Classifier**	m7	m9	m4	m1	m5	m5	m4	m6	m4, m5
	**Value**	0.32	0.422	0.533	0.576	0.476	0.684	0.43	0.452	
**Tpr 7**	**Classifier**	m5	m5	m5	m6	m10	m6	m1	m9	m5
	**Value**	0.73	0.47	0.678	0.483	0.844	0.768	0.54	0.721	
**Best classifier/option-Tpr parameter**		m7	m5	m2, m4, m5, m10	m6, m10	m5	m6	m1	m6, m8	

The best classifiers and their values are 5xMCCV5, depending on the option for the accuracy balanced (Acc balanced) parameter and the best classifiers for the true positive rate parameter according to individual classes (Tpr 1, Tpr 5, Tpr 6, and Tpr 7). As a summary, the classifiers that most often gave the best results for a given parameter (column Best classifier/parameter) and options (row Best classifier/option, TPR parameter) were given.

**Table 4:** Average classification result from 25 tests, 5xMCCV5 experiment, 7 vs. all, M1 device, wooden chamber (Option I). Classifiers: m1 = canberra.1nn, m2 = canberra.811, m3 = eps = 0.01.nb, m4 = eps = 0.01.nb2, m5 = euclidean.1nn, m6 = euclidean.811, m7 = manhattan.1nn, m8 = manhattan.811, m9 = maxminnormalized.1nn, and m10 = maxminnormalized.811.

Classifier	acc _global_	cov _global_	acc _156_	acc _7_	acc _balanced_	tpr _156_	tpr _7_
m1	0.804204	1	0.941696	0.421144	0.681416	0.831952	0.718668
m2	0.59578	1	0.46676	1	0.73338	1	0.386401
m3	0.639988	1	0.436116	0.57086	0.631352	0.831816	0.385934
m4	0.522104	1	0.930472	0.838284	0.6372	0.903864	0.331623
m5	0.835788	1	0.930472	0.59462	0.762544	0.871184	0.742336
m6	0.66526	1	0.589488	0.892668	0.741072	0.962496	0.42656
m7	0.802104	1	0.928148	0.488048	0.708096	0.840752	0.637808
m8	0.61894	1	0.50308	0.968668	0.73588	0.989336	0.395088
m9	0.776836	1	0.941556	0.324333	0.632944	0.806192	0.580668
m10	0.59578	1	0.474112	0.978668	0.726392	0.989848	0.38302

**Table 5:** Average classification result from 25 tests, 5xMCCV5 experiment, 7 vs. all, M1 device, polystyrene chamber (Option II). Classifiers: m1 = uclidea.1nn, m2 = uclidea.811, m3 = eps = 0.01.nb, m4 = eps = 0.01.nb2, m5 = uclidean.1nn, m6 = uclidean.811, m7 = manhattan.1nn, m8 = manhattan.811, m9 = maxminnormalized.1nn, and m10 = maxminnormalized.811.

Classifier	acc _global_	cov _global_	acc _156_	acc _7_	acc _balanced_	tpr _156_	tpr _7_
m1	0.757892	1	0.881348	0.434096	0.65772	0.808972	0.589808
m2	0.469476	1	0.408336	0.65762	0.532972	0.673972	0.302018
m3	0.635784	1	0.818772	0.157048	0.487912	0.726604	0.195856
m4	0.477892	1	0.471108	0.51562	0.493364	0.737268	0.250017
m5	0.766312	1	0.858332	0.53524	0.696788	0.834828	0.608952
m6	0.50316	1	0.409024	0.763432	0.586228	0.808416	0.360549
m7	0.732632	1	0.841324	0.45848	0.649904	0.809468	0.527712
m8	0.4779	1	0.391062	0.720764	0.555908	0.749288	0.32875
m9	0.741056	1	0.85872	0.434096	0.646404	0.804592	0.515332
m10	0.503164	1	0.467176	0.603336	0.535252	0.739672	0.326092

**Table 6:** Average classification result from 25 tests, 5xMCCV5 experiment, 7 vs. all, M1 device, wooden chamber, baseline correction (Option V). Classifiers: m1 = canberra.1nn, m2 = canberra.811, m3 = eps = 0.01.nb, m4 = eps = 0.01.nb2, m5 = euclidean.1nn, m6 = euclidean.811, m7 = manhattan.1nn, m8 = manhattan.811, m9 = maxminnormalized.1nn, and m10 = maxminnormalized.811.

Classifier	acc _global_	cov _global_	acc _156_	acc _7_	acc _balanced_	tpr _156_	tpr _7_
m1	0.770524	1	0.944852	0.347047	0.645948	0.790696	0.622
m2	0.604212	1	0.465928	0.984	0.724968	0.994668	0.404204
m3	0.621048	1	0.692736	0.524856	0.6088	0.795492	0.367568
m4	0.543152	1	0.469512	0.804	0.636764	0.867452	0.341942
m5	0.80842	1	0.934388	0.51124	0.722808	0.837764	0.710188
m6	0.669476	1	0.592276	0.881712	0.736984	0.943892	0.450932
m7	0.778948	1	0.942352	0.383333	0.662844	0.800552	0.654
m8	0.658944	1	0.543292	0.978284	0.76078	0.990224	0.442968
m9	0.755788	1	0.938508	0.306285	0.622388	0.780056	0.562004
m10	0.610528	1	0.485748	0.957904	0.721832	0.980056	0.403856

**Table 7:** Average classification result from 25 tests, 5xMCCV5 experiment, 7 vs. all, M1 device, polystyrene chamber, baseline correction (Option VI). Classifiers: m1 = canberra.1nn, m2 = canberra.811, m3 = eps = 0.01.nb, m4 = eps = 0.01.nb2, m5 = euclidean.1nn, m6 = euclidean.811, m7 = manhattan.1nn, m8 = manhattan.811, m9 = maxminnormalized.1nn, and m10 = maxminnormalized.811.

Classifier	acc _global_	cov _global_	acc _156_	acc _7_	acc _balanced_	tpr _156_	tpr _7_
m1	0.732624	1	0.850236	0.454	0.65212	0.798528	0.594572
m2	0.59158	1	0.447148	0.975	0.711076	0.986668	0.428288
m3	0.654732	1	0.826652	0.253952	0.540308	0.735512	0.396762
m4	0.532628	1	0.483588	0.67662	0.580104	0.786232	0.342526
m5	0.751572	1	0.843516	0.55976	0.70164	0.833768	0.559636
m6	0.738944	1	0.85024	0.450952	0.6506	0.81728	0.650192
m7	0.74526	1	0.820408	0.588192	0.704296	0.840516	0.588724
m8	0.730524	1	0.810488	0.540284	0.675384	0.832588	0.589084
m9	0.741048	1	0.85594	0.469048	0.662496	0.803396	0.612092
m10	0.71368	1	0.694484	0.789996	0.74224	0.915252	0.5277

**Table 8:** Classes 1, 5, 6, and 7 vs. all—true positive rate; best classifiers, 5xMCCV5—average classification results from 25 tests: all classes, M1 and M2 devices, wooden and polystyrene chambers, with and without baseline correction (Options I–VIII). Note: Tpr 1 = true positive rate class 1, Tpr 5 = true positive rate class 5, Tpr 6 = true positive rate class 6, and Tpr 7 = true positive rate class 7.

		Option I	Option II	Option III	Option IV	Option V	Option VI	Option VII	Option VIII	Best Classifier/ parameter
**Tpr 1 vs. 5, 6, 7**	**Classifier**	m2	m5	m2	m2, m5, m6, m7, m10	m2, m10	m2	m1	m1, m9	m2
	**Value**	0.981	0.817	0.948	1	0.975	0.995	0.902	1	
**Tpr 5 vs. 1, 6, 7**	**Classifier**	m6	m5	m6	m6	m6	m10	m1	m6	m6
	**Value**	0.544	0.55	0.51	0.51	0.525	0.284	0.582	0.530	
**Tpr 6 vs. 1, 5, 7**	**Classifier**	m5	m9	m1	m1	m5	m6	m5	m1, m9	m1, m5
	**Value**	0.532	0.514	0.489	0.563	0.372	0.619	0.542	0.413	
**Tpr 7 vs. 1, 5, 6**	**Classifier**	m5	m5	m5	m5	m5	m5	m9	m5	m5
	**Value**	0.742	0.61	0.417	0.376	0.710	0.650	0.427	0.489	
**Best classifier/Option**		m5	m5	m1, m2, m5, m6	m5, m6	m5	m2, m5, m6, m10	m1	m1, m9	

The best classifiers and their results depending on the option for the true positive rate parameter in tests 1 vs. all performed for each class. As a summary, the classifiers delivering the best results for a given parameter (column Best classifier/parameter) and option (row Best classifier/option) were given. In individual options, the m5 classifier appears as the best (or equivalent) most frequently. In the case of parameters, the m5 classifier also appears most often (twice: in test 6 vs. 1, 5, 7 and 7 vs. 1, 5, 6).

**Table 9:** Classes 1, 5, 6, and 7 vs. all—accuracy balanced; best classifiers, 5xMCCV5—average classification results from 25 tests: all classes, M1 and M2 devices, wooden and polystyrene chambers, with and without baseline correction (Options I–VIII).

		Option I	Option II	Option III	Option IV	Option V	Option VI	Option VII	Option VIII	Best Classifier/ Parameter
**Acc _balanced_ 1 vs. 5, 6, 7**	**Classifier**	m7	m9	m10	m6	m10	m9	m1	m1	m1, m9, m10
	**Value**	0.864	0.788	0.887	0.925	0.866	0.886	0.896	0.964	
**Acc _balanced_ 5 vs. 1, 6, 7**	**Classifier**	m6	m5	m6	m10	m6	m10	m1	m6	m6
	**Value**	0.752	0.741	0.725	0.663	0.713	0.638	0.736	0.707	
**Acc _balanced_ 6 vs. 1, 5, 7**	**Classifier**	m6	m5	m10	m1	m10	m5	m10	m10	m10
	**Value**	0.819	0.814	0.758	0.822	0.652	0.775	0.792	0.697	
**Acc _balanced_ 7 vs. 1, 5, 6**	**Classifier**	m5	m5	m5	m5	m1	m10	m9	m5	m5
	**Value**	0.763	0.697	0.652	0.665	0.696	0.742	0.427	0.656	
**Best classifier/ option**		m6	m5	m10	m1, m5, m6, m10	m10	m10	m1	m1, m5, m6, m10	

The best classifiers depending on the options for the accuracy balanced parameter in tests type 1 vs. all performed for each class. As a summary, the classifiers delivering the best results for a given parameter (column Best classifier/parameter) and option (row Best classifier/option) were given. In individual options, the m5 classifier appears as the best (or equivalent) most frequently. In the case of parameters, the m5 classifier also appears most often (twice: in test 6 vs. 1, 5, 7 and 7 vs. 1, 5, 6).

**Table 10:** Class 7 vs. 1, 5, 6—accuracy balanced; true positive rate, results of separation. Results in options for the m5, euclidean.1nn method—the most effective method for distinguishing class 7; comparison of options, analysis of baseline correction application.

	Option I	Option II	Option III	Option IV	Option V	Option VI	Option VII	Option VIII
**Acc _balanced_ True positive rate class 7**	0.763	0.697	0.651	0.558	0.723	0.702	0.612	0.601
	0.742	0.609	0.417	0.36	0.612	0.56	0.349	0.47

## Data Availability

The data presented in this study are available on request from the corresponding author. The data are not publicly available due to business secret.

## References

[1] Van Zweden J.S., D’Ettorre P., Blomquist G.J., Bagneres A.-G. (2010). Nestmate recognition in social insects and the role of hydrocarbons. Insect Hydrocarbons.

[2] Vernier C., Krupp J., Marcus K., Hefetz A., Levine J., Ben-Shahar Y. (2019). The cuticular hydrocarbon profiles of honey bee workers develop via a socially-modulated innate process. eLife.

[3] Butler C.G. (1959). Queen Substance. Bee World.

[4] Hoover S., Keeling C., Winston M., Slessor K. (2003). The effect of queen pheromones on worker honey bee ovary development. Naturwissenschaften.

[5] Rojek W., Kuszewska K., Ostap-Chęć M., Woyciechowski M. (2019). Do rebel workers in the honeybee Apis mellifera avoid worker policing?. Apidologie.

[6] Gregg A.L. (1939). The Unexplained Queen. Bee World.

[7] Woyke J. (1963). Drones from fertilized eggs and biology of sex determination in the honeybee. Bull. Acad. Polon. Sci. Cl..

[8] Gardner J.W., Bartlett P.N. (1994). A brief history of electronic noses. Sens. Actuators B Chem..

[9] Dymerski T., Gebicki J., Wardencki W., Namieśnik J. (2014). Application of an Electronic Nose Instrument to Fast Classification of Polish Honey Types. Sensors.

[10] Szczurek A., Maciejewska M., Bąk B., Wilk J., Wilde J., Siuda M. (2020). Gas sensor array and classifier as a means of varroosis detection. Sensors.

[11] Schaller E., Bosset J., Escher F. (1998). “Electronic Noses” and Their Application to Food. LWT Food Sci. Technol..

[12] Benedetti S., Mannino S., Sabatini A.G., Marcazzan G.L. (2004). Original article electronic nose and neural network use for the classification of honey. Apidologie.

[13] Ampuero S., Bogdanov S., Bosset J.O. (2004). Classification of unifloral honeys with an MS-based electronic nose using different sampling modes: SHS, SPME and INDEX. Eur. Food Res. Technol..

[14] Abellán J., Castellano J.G. (2017). Improving the Naive Bayes Classifier via a Quick Variable Selection Method Using Maximum of Entropy. Entropy.

[15] Saadatfar H., Khosravi S., Joloudari J.H., Mosavi A., Shamshirband S. (2020). A New K-Nearest Neighbors Classifier for Big Data Based on Efficient Data Pruning. Mathematics.

[16] Szczurek A., Maciejewska M., Bąk B., Wilde J., Siuda M. (2019). Semiconductor gas sensor as a detector of Varroa destructor infestation of honeybee colonies—Statistical evaluation. Comput. Electron. Agric..

[17] Polkowski L., Artiemjew P. (2015). Granular Computing in Decision Approximation, An Application of Rough Mereology. Series: Intelligent Systems Reference Library.

